# Supplementary material for: An environment for sustainable research software in Germany and beyond: current state, open challenges, and call for action
Source: F1000Res. 2021 Jan 26;9:295. Originally published 2020 Apr 27. [Version 2] doi: 10.12688/f1000research.23224.2 (PMC7845155; doi:10.12688/f1000research.23224.2)
Supplement: Peer review history

# Peer review: Reviewer response for version 2

**DOI:** 10.5256/f1000research.31504.r78211

**Published:** 2021-01-28

**Authors:** Willi Hasselbring

**Affiliations:** 1 Software Engineering Group, Kiel University, Kiel, Germany

Thanks for delivering this revised version of your opinion article. I highly appreciate that you addressed all the concerns I had with the previous version, such that I can now fully approve your paper!

Is the topic of the opinion article discussed accurately in the context of the current literature?

Yes

Are arguments sufficiently supported by evidence from the published literature?

Partly

Are all factual statements correct and adequately supported by citations?

Yes

Are the conclusions drawn balanced and justified on the basis of the presented arguments?

Partly

Reviewer Expertise:

Software Engineering

I confirm that I have read this submission and believe that I have an appropriate level of expertise to confirm that it is of an acceptable scientific standard.

# Peer review: Reviewer response for version 1

**DOI:** 10.5256/f1000research.25640.r62872

**Published:** 2020-06-01

**Authors:** Radovan Bast

**Affiliations:** 1 Department of Information Technology, UiT The Arctic University of Norway, Tromsø, Norway

In "An environment for sustainable research software in Germany and beyond: current state, open challenges, and call for action" the authors identify challenges for research software sustainability in Germany and beyond.

 They examine the current state of research software sustainability and challenges in motivating sustainable research software development, selection criteria for funding, personnel, funding, infrastructure, and legal aspects, and offer recommendations for addressing these challenges. These sections are accompanied and with a story using a fictional character Kim which helps to

 relate these aspects to typical career stages of a research software engineer.

 The article is thoroughly researched, well-written, and offers an excellent overview of the challenges when building an environment for sustainable research software. Most of the discussed challenges and recommendations carry beyond Germany and are relevant and transferable to other countries.

 Below I give few (minor) suggestions for consideration when improving the manuscript.

 Regarding the list of challenges under "Why sustainable research software in the first place?" (pages 5 and 6):

- Infrastructure issues: One design choice that often limits the use or usability of local infrastructure resources is that they are often bound to institutional user accounts and thus limit collaboration possibilities with collaborators in other institutions and countries. On the other hand, pooling of infrastructure resources which could enable collaboration across organizations can be limited by lack of authentication and authorization infrastructure (AAI) or legal constraints. Later in the paper the authors indeed mention AAI (page 12) but this could already be pointed out and connected earlier.
- Legal issues: Not only licensing is an issue but legal constraints or uncertainty about legal boundaries and identity federation can also limit the deployment of infrastructure services. Often the deployment and operation of infrastructure services is given to technical teams who may lack the legal support or expertise to clarify legal and privacy terms for the storage of data and processing of data.
- Funding issues: The challenge is not only that funding is scarce but also that it does not align well with pricing models of cloud infrastructure providers. It can be easier for research groups to spend a larger chunk of the budget towards the end of a year for hardware compared to pay possibly relatively modest monthly fees for a cloud service, which however may not fit into the budget forms. These budget constraints may also limit the possibility of pooling resources and sharing them with other research groups. Software cloud infrastructure is often not considered at all in the proposal. There is also a resistance among some of my research colleagues to pay 20-50 USD/ month for an infrastructure service which is sometimes solved by reinventing the service locally "for free".
- Another mismatch between traditional funding models and support of software which "must continue to exist" to be sustainable (page 5), is the experience that it can take months or years until the software is picked up by other groups and contributions and questions start to roll in. But by that time the funding of the project stopped, the developer (team) may have already moved on to other positions and projects, and may not have the time to react and help, even though they still may have interest and the knowledge. Our traditional funding models consider the software to be "done" by the end of the project.

Selection criteria for "How to decide which software to sustain?" (page 9):

- The authors mention "usage and impact", "software quality", as well as "maturity". But I would like to see also "openness and transparency" among these. The reason is that we can expect the research community to adapt to these or any metrics and we will over time observe what we measure. Any set of metrics could be criticized as to some extent being arbitrary but the advantage of including "openness and transparency" is that the community as whole would benefit from such a metric [Enrico Glerean, "Responsible conduct of research and questionable research practices", presentation, slide 47 ^1^].

Regarding "Who sustains research software?":

- The authors discuss the lack of recognition and awareness, as well as lack of career opportunities. It is also about respect and I was happy to see the sentence: "Not all researchers that think of themselves as RSEs pursue a faculty position as their main career goal." I have experienced that RSEs are sometimes regarded as those who somehow "failed" to obtain a faculty position whereas many RSEs have chosen this position over a faculty position because it was a better fit for their career goals. This misunderstanding can lead to a lack of respect towards this position and this career choice and can lead to excellent personnel leaving the academic environment towards commercial employment, possibly not primarily for financial reasons but sometimes to be more respected and recognized.

Archiving and software preservation (page 13:

- The authors mention Docker but also Singularity should be mentioned as a tool since it is getting traction in particular on many-user systems such as higher performance computing clusters.

Legal aspects (page 14):

- Re-licensing is mentioned in the story box and the text starts by pointing out that licensing is often not considered early enough in the project. Indeed re-licensing later in the project can be not only legally, but also organizationally very tricky, in particular for projects which developed over many years and involved many contributors in different organizations. This could be pointed out in the text as additional motivation to consider these very early in the project.
- I very much like the recommendation of providing a legal help desk for research groups to avoid the problem that out of uncertainty and fear of making a legal mistake some research groups end up not choosing any license at all which may limit further reuse of the software.
- The manuscript presents a decision tree for contributors (Figure 1) and also discusses contributor license agreements. It could be useful to point out that without clear policies or legal help desks, individuals or organizations may be hesitant to contribute to a project because they may not feel confident having enough knowledge or authority to sign such agreements and too many legal steps and question can also raise the barrier to contribute, in particular for smaller projects. Also here clear guidelines and a support desk can help removing these barriers.

Is the topic of the opinion article discussed accurately in the context of the current literature?

Yes

Are arguments sufficiently supported by evidence from the published literature?

Yes

Are all factual statements correct and adequately supported by citations?

Yes

Are the conclusions drawn balanced and justified on the basis of the presented arguments?

Yes

Reviewer Expertise:

computational chemistry, research software engineering

I confirm that I have read this submission and believe that I have an appropriate level of expertise to confirm that it is of an acceptable scientific standard.

## References

[1] : Responsible conduct of research and questionable research practices.2018; 10.6084/m9.figshare.10303487.v1

# Response

**Published:** 2020-12-17

**Authors:** Axel Loewe

**Affiliations:** Karlsruhe Institute of Technology, Germany

*We thank you for the thorough review and constructive feedback regarding the manuscript. Below, we address the issues raised by you point-by-point. Our responses are set in italics.*

**Regarding the list of challenges under "Why sustainable research software in the first place?" (pages 5 and 6):**

 Infrastructure issues: One design choice that often limits the use or usability of local infrastructure resources is that they are often bound to institutional user accounts and thus limit collaboration possibilities with collaborators in other institutions and countries. On the other hand, pooling of infrastructure resources which could enable collaboration across organizations can be limited by lack of authentication and authorization infrastructure (AAI) or legal constraints. Later in the paper the authors indeed mention AAI (page 12) but this could already be pointed out and connected earlier.

*We thank the reviewer for their suggestion, and have included the mentioned issues in the respective list in the section “Why sustainable research software in the first place?”.*

****

 Legal issues: Not only licensing is an issue but legal constraints or uncertainty about legal boundaries and identity federation can also limit the deployment of infrastructure services. Often the deployment and operation of infrastructure services is given to technical teams who may lack the legal support or expertise to clarify legal and privacy terms for the storage of data and processing of data.

*We thank the reviewer for their suggestion, and have included the mentioned issues in the respective list in the section “Why sustainable research software in the first place?”*

**

 Funding issues: The challenge is not only that funding is scarce but also that it does not align well with pricing models of cloud infrastructure providers. It can be easier for research groups to spend a larger chunk of the budget towards the end of a year for hardware compared to pay possibly relatively modest monthly fees for a cloud service, which however may not fit into the budget forms. These budget constraints may also limit the possibility of pooling resources and sharing them with other research groups. Software cloud infrastructure is often not considered at all in the proposal. There is also a resistance among some of my research colleagues to pay 20-50 USD/ month for an infrastructure service which is sometimes solved by reinventing the service locally "for free".

 Another mismatch between traditional funding models and support of software which "must continue to exist" to be sustainable (page 5), is the experience that it can take months or years until the software is picked up by other groups and contributions and questions start to roll in. But by that time the funding of the project stopped, the developer (team) may have already moved on to other positions and projects, and may not have the time to react and help, even though they still may have interest and the knowledge. Our traditional funding models consider the software to be "done" by the end of the project.

*We thank the reviewer for these two comments. To address them, we have extended the “Funding issues” list item with a discussion of these issues.*

**Selection criteria for "How to decide which software to sustain?" (page 9):**

 The authors mention "usage and impact", "software quality", as well as "maturity". But I would like to see also "openness and transparency" among these. The reason is that we can expect the research community to adapt to these or any metrics and we will over time observe what we measure. Any set of metrics could be criticized as to some extent being arbitrary but the advantage of including "openness and transparency" is that the community as whole would benefit from such a metric [Enrico Glerean, "Responsible conduct of research and questionable research practices", presentation, slide 471].

*We thank the reviewer for this suggestion and realized that indeed most aspects in this section (6-10, 12, 13) are actually related to openness and transparency. Therefore, we changed the title of this section to “Software transparency and quality”.*

**Regarding "Who sustains research software?":**

 The authors discuss the lack of recognition and awareness, as well as lack of career opportunities. It is also about respect and I was happy to see the sentence: "Not all researchers that think of themselves as RSEs pursue a faculty position as their main career goal." I have experienced that RSEs are sometimes regarded as those who somehow "failed" to obtain a faculty position whereas many RSEs have chosen this position over a faculty position because it was a better fit for their career goals. This misunderstanding can lead to a lack of respect towards this position and this career choice and can lead to excellent personnel leaving the academic environment towards commercial employment, possibly not primarily for financial reasons but sometimes to be more respected and recognized.

*We fully agree with the reviewer and thank them for the renewed confirmation that this is seen as problematic not only by the authors.*

**Archiving and software preservation (page 13):**

 The authors mention Docker but also Singularity should be mentioned as a tool since it is getting traction in particular on many-user systems such as higher performance computing clusters.

*We thank the reviewer for this suggestion and now also mention Singularity and GUIX. However, we are not aiming for an exhaustive list, as options change dynamically and might even be specific to certain research communities.*

**Legal aspects (page 14):**

 Re-licensing is mentioned in the story box and the text starts by pointing out that licensing is often not considered early enough in the project. Indeed re-licensing later in the project can be not only legally, but also organizationally very tricky, in particular for projects which developed over many years and involved many contributors in different organizations. This could be pointed out in the text as additional motivation to consider these very early in the project.

*We thank the reviewer to point this out and it also nicely fits into the message of increasing the awareness of legal aspects early on in the project. We have added the suggested sentence in the manuscript.*

****

 I very much like the recommendation of providing a legal help desk for research groups to avoid the problem that out of uncertainty and fear of making a legal mistake some research groups end up not choosing any license at all which may limit further reuse of the software.

*Thank you for supporting this idea. We have further included your idea of avoiding any license out of a fear to make legal mistakes.*

****

 The manuscript presents a decision tree for contributors (Figure 1) and also discusses contributor license agreements. It could be useful to point out that without clear policies or legal help desks, individuals or organizations may be hesitant to contribute to a project because they may not feel confident having enough knowledge or authority to sign such agreements and too many legal steps and question can also raise the barrier to contribute, in particular for smaller projects. Also here clear guidelines and a support desk can help removing these barriers.

*We have decided to take the decision trees out of the manuscript to strengthen our point of publishing software under a FLOSS license. Instead, we published the decision trees together with documentation templates under a Creative Commons license via Zenodo: https://zenodo.org/record/4327148#.X9n6ui337OQ. In order to strengthen the point you addressed, we added some more details related to infrastructural investment.*

# Peer review: Reviewer response for version 1

**DOI:** 10.5256/f1000research.25640.r62873

**Published:** 2020-05-13

**Authors:** Willi Hasselbring

**Affiliations:** 1 Software Engineering Group, Kiel University, Kiel, Germany

The authors describe the state of the practice and current challenges for research software sustainability and suggest measures towards improvements that can solve these challenges. In particular, they propose to fund a German Software Sustainability Institute. The paper is the result of a community effort, with work undertaken during two workshops and subsequent collaborative work across the larger RSE community in Germany.

 The UK Software Sustainability Institute has already been established during a decade ( https://www.software.ac.uk/blog/2020-05-05-impact-institute-10-years). Thus, the idea of such an institute is not new, but it makes sense to take a specific look at the German situation. Besides universities, the German states (local and in particular federal) fund significant large-scale research associations (Helmholtz/DLR, Max-Planck, Leibniz). This is not the case for most other European states, at least not with a similar scale. Another specialty is the lack of long-term funding for research software engineers, as discussed by the authors.

 The paper is well-written and easy to read. I like the boxed story of Kim’s career path.

 However, I’ve some suggestions for improving the paper:

- Concerning the statement “In order to support research, a sustainable software must be correct”, I suggest to include a short discussion of the test oracle problem for scientific software (see for instance https://doi.org/10.1109/SECSE.2013.6615099 ^1^).
- Concerning the discussion of “The list of criteria presented in this section could be the basis for a structured review process…” I suggest to include two additional initiatives for software review. The first is artifact evaluation in computer science conferences (the process is explained in https://doi.org/10.1515/itit-2019-0040 ^2^). The second is the SPEC Research Group’s review process of tools for quantitative system evaluation and analysis (https://research.spec.org/tools/submission.html).

The authors write “We also argue that truly sustainable research software must ideally be published under a Free/Libre Open Source Software (FLOSS) license, and follow an open development model…” what I fully support (see for instance https://doi.org/10.1515/itit-2019-0040 ^2^). However, later under the section heading “Legal aspects” this requirement is thwarted. I fully agree that legal aspects have to be considered, but the general bias of this section seems to be on commercial licensing of research software. For instance, the decision tree in Figure 1 starts with the question “Licensing planned?”. I assume that commercial licensing is meant, but this is not clear since the figures are not explained in the paper. Instead, the process should start with open sourcing the software. If licenses such as Apache or MIT are applied, the research institutions may later still commercialize the software if appropriate. Such open source licensing is also beneficial for start-ups, that intend to provide professional services for the software.

 My experience with technology transfer units of German universities and research institutes is that they do not understand the ideas of open source business models (see for instance https://doi.org/10.1109/MC.2019.2898163 ^3^). Their focus is on patents and commercializing licenses, sometimes also on start-ups. Conversely, in the software industry, one major motivation for open sourcing software is on improving the quality of software. I cite from https://doi.org/10.1109/ICSAW.2017.11 ^4^ : “the open-source approach has some psychological effects: Developers show a tendency to apply higher quality standards if they know that the code will be publicly available.” For sustainability, quality is an important property of software.

 The Figures 1-4 do more harm than good. They are daunting to researchers who intend to publish their code open source. These figures should be removed from the paper, they are useless without proper explanation.

 I suggest that the authors focus in the present paper on their main message (request for funding a German Software Sustainability Institute, which I fully support). Figures 1-4 could be moved to a separate paper, enriched with proper explanation.

Is the topic of the opinion article discussed accurately in the context of the current literature?

Yes

Are arguments sufficiently supported by evidence from the published literature?

Partly

Are all factual statements correct and adequately supported by citations?

Yes

Are the conclusions drawn balanced and justified on the basis of the presented arguments?

Partly

Reviewer Expertise:

Software Engineering

I confirm that I have read this submission and believe that I have an appropriate level of expertise to confirm that it is of an acceptable scientific standard, however I have significant reservations, as outlined above.

## References

[1] : Techniques for testing scientific programs without an oracle. *IEEE Xplore*.2013; 10.1109/SECSE.2013.6615099 10.1109/SECSE.2013.6615099

[2] : From FAIR research data toward FAIR and open research software. *it - Information Technology*.2020;62(1) : 10.1515/itit-2019-0040 39-47 10.1515/itit-2019-0040

[3] : The Innovations of Open Source. *Computer*.2019;52(4) : 10.1109/MC.2019.2898163 59-63 10.1109/MC.2019.2898163

[4] : Microservice Architectures for Scalability, Agility and Reliability in E-Commerce. *IEEE*.2017; 10.1109/ICSAW.2017.11 10.1109/ICSAW.2017.11

[5] Tools Submission Portal. *SPEC Research Group*. Reference source

# Response

**Published:** 2020-12-17

**Authors:** Axel Loewe

**Affiliations:** Karlsruhe Institute of Technology, Germany

*Thank you for the thorough review and constructive feedback regarding our manuscript. Below, we address the issues raised by you point-by-point. Our responses are set in italics.*

 Concerning the statement “In order to support research, a sustainable software must be correct”, I suggest to include a short discussion of the test oracle problem for scientific software (see for instance https://doi.org/10.1109/SECSE.2013.66150991).

*We thank the reviewer for this suggestion, and included a brief discussion of the test oracle problem as suggested, and additionally of further challenges to verification and validation, such as large configuration spaces and heterogeneous data (as discussed in e.g. https://doi.org/10.1109/SE4Science.2019.00010), and have suggested to implement the solutions mentioned in the literature.*

 Concerning the discussion of “The list of criteria presented in this section could be the basis for a structured review process…” I suggest to include two additional initiatives for software review. The first is artifact evaluation in computer science conferences (the process is explained in https://doi.org/10.1515/itit-2019-00402). The second is the SPEC Research Group’s review process of tools for quantitative system evaluation and analysis ( https://research.spec.org/tools/submission.html).

*We thank the reviewer for this suggestion and included the artifact review approach in the introduction to the criteria section. The aspects of repeatability, reproducibility, and replicability are aimed more at the results of computational research rather than research software itself, we feel. Therefore, we didn’t include specific criteria in the list suggested to be used when evaluating research software for sustained funding.*

*While the SPEC submission process is very clear, we could not find any concrete criteria applied during the review (except for requirements regarding the license).*

 The authors write “We also argue that truly sustainable research software must ideally be published under a Free/Libre Open Source Software (FLOSS) license, and follow an open development model…” what I fully support (see for instance https://doi.org/10.1515/itit-2019-00402). However, later under the section heading “Legal aspects” this requirement is thwarted. I fully agree that legal aspects have to be considered, but the general bias of this section seems to be on commercial licensing of research software. For instance, the decision tree in Figure 1 starts with the question “Licensing planned?”. I assume that commercial licensing is meant, but this is not clear since the figures are not explained in the paper. Instead, the process should start with open sourcing the software. If licenses such as Apache or MIT are applied, the research institutions may later still commercialize the software if appropriate. Such open source licensing is also beneficial for start-ups that intend to provide professional services for the software. 

 My experience with technology transfer units of German universities and research institutes is that they do not understand the ideas of open source business models (see for instance https://doi.org/10.1109/MC.2019.28981633). Their focus is on patents and commercializing licenses, sometimes also on start-ups. Conversely, in the software industry, one major motivation for open sourcing software is on improving the quality of software. I cite from https://doi.org/10.1109/ICSAW.2017.114 : “the open-source approach has some psychological effects: Developers show a tendency to apply higher quality standards if they know that the code will be publicly available.” For sustainability, quality is an important property of software.

*As further detailed below, we have moved the decision trees out of this manuscript as we see the problems and agree to your arguments. We like your suggested aspect of commercialization of FLOSS licensed software and included this aspect in the manuscript.*

 The Figures 1-4 do more harm than good. They are daunting to researchers who intend to publish their code open source. These figures should be removed from the paper, they are useless without proper explanation.

 I suggest that the authors focus in the present paper on their main message (request for funding a German Software Sustainability Institute, which I fully support). Figures 1-4 could be moved to a separate paper, enriched with proper explanation.

*We thank the reviewer for this suggestion. Our initial thought was to place these decision trees in the supplemental material but did not realize that this is not the policy of f1000. The editorial team moved them into the main article. This is the reason why the decision trees appeared without additional information in the manuscript. We have now decided to take the Figures out and have published them together with documentation templates in a separate report via Zenodo unde a Creative Commons license, see https://zenodo.org/record/4327148#.X9n6ui337OQ. This report is now cited in the F1000 manuscript.*
